# Proteomic analysis reveals potential therapeutic targets for childhood asthma through Mendelian randomization

**DOI:** 10.1002/clt2.12357

**Published:** 2024-05-10

**Authors:** Yi‐Qing Wu, Yi‐Xin Cai, Xiao‐Li Chen, Shang‐Qin Chen, Xiu‐Feng Huang, Zhen‐Lang Lin

**Affiliations:** ^1^ Department of Pediatrics The Second Affiliated Hospital and Yuying Children's Hospital of Wenzhou Medical University Wenzhou Zhejiang China; ^2^ The Second School of Medicine Wenzhou Medical University Wenzhou Zhejiang China; ^3^ Key Laboratory of Perinatal Medicine of Wenzhou Wenzhou Zhejiang China; ^4^ Key Laboratory of Structural Malformations in Children of Zhejiang Province Wenzhou Zhejiang China; ^5^ Zhejiang Provincial Clinical Research Center for Pediatric Disease The Second Affiliated Hospital and Yuying Children's Hospital of Wenzhou Medical University Wenzhou Zhejiang China

**Keywords:** childhood asthma, Mendelian randomization, plasma proteome, risk factors, therapeutic targets

## Abstract

**Background:**

Asthma is the most common chronic disease among children and poses a significant threat to their health. This study aims to assess the relationship between various plasma proteins and childhood asthma, thereby identifying potential therapeutic targets.

**Methods:**

Based on publicly available genome‐wide association study summary statistics, we employed a two‐sample Mendelian randomization (MR) approach to elucidate the causal relationship between plasma proteins and asthma. Mediation analysis was then conducted to evaluate the indirect influence of plasma proteins on childhood asthma mediated through risk factors. Comprehensive analysis was also conducted to explore the association between plasma proteins and various phenotypes using the UK Biobank dataset.

**Results:**

MR analysis uncovered a causal relationship between 10 plasma proteins and childhood asthma. Elevated levels of seven proteins (TLR4, UBP25, CBR1, Rac GTPase‐activating protein 1 [RGAP1], IL‐21, MICB, and PDE4D) and decreased levels of three proteins (GSTO1, LIRB4 and PIGF) were associated with an increased risk of childhood asthma. Our findings further validated the connections between reported risk factors (body mass index, mood swings, hay fever or allergic rhinitis, and eczema or dermatitis) and childhood asthma. Mediation analysis revealed the influence of proteins on childhood asthma outcomes through risk factors. Furthermore, the MR analysis identified 73 plasma proteins that exhibited causal associations with at least one risk factor for childhood asthma. Among them, RGAP1 mediates a significant proportion (25.10%) of the risk of childhood asthma through eczema or dermatitis. Finally, a phenotype‐wide association study based on these 10 proteins and 1403 diseases provided novel associations between these biomarkers and multiple phenotypes.

**Conclusion:**

Our study comprehensively investigated the causal relationship between plasma proteins and childhood asthma, providing novel insights into potential therapeutic targets.

## 1 INTRODUCTION

Asthma is the most common chronic respiratory disease in children with wheezing, coughing and dyspnea as the main symptoms, and its global prevalence in children is as high as 14.1%, seriously endangering children's health.^1^, ^2^, ^3^ Therefore, proper prevention, early diagnosis and reasonable treatment are especially essential. Various risk factors can influence asthma development, and their effects may vary among individuals at different stages of growth and development.^4^, ^5^ Nevertheless, further research is warranted to explore the risk factors and treatment options associated with asthma development in both pediatric and adult populations. Plasma proteins play a crucial role in the onset and progression of diseases, serving as valuable tools to identify potential diagnostic biomarkers.^6^, ^7^, ^8^, ^9^ Notably, plasma proteins are closely associated with allergic diseases such as asthma.^10^, ^11^

Genetic variants associated with plasma protein levels have been identified by genome‐wide association studies (GWAS) and are defined as “protein quantitative trait loci (pQTLs).”^12^, ^13^ PQTLs provide an opportunity to utilize Mendelian randomization (MR) methods in investigating the causal effects of potential drug targets on human disease phenotypes.^14^, ^15^ MR is an effective method employed for causal inference,^16^ using genetic variation as an instrumental variable (IV) to ascertain causal relationships between exposures (e.g., plasma proteins) and outcomes (e.g., childhood asthma), effectively avoiding the confounding bias of traditional epidemiological studies.^17^

In order to explore the causal relationship between plasma proteins and childhood asthma, we initially performed a two‐sample MR analysis using genetic IVs.^18^ Subsequently, we identified risk factors associated with childhood asthma and evaluated the causal relationship between plasma proteins and these risk factors.^19^ Additionally, we conducted a mediation analysis to evaluate the indirect impact of plasma proteins on childhood asthma mediated by the presence of risk factors. Finally, we performed a phenotype‐wide association study analysis using the UK Biobank dataset to assess the suitability of targeting these proteins for the treatment of childhood asthma.

## 2 METHODS

### 2.1 Study design

**FIGURE 1:**
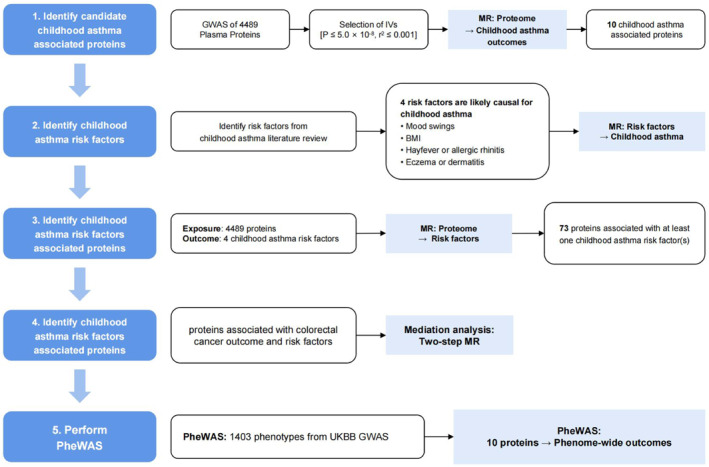
Overview of MR analyses. This study employed two‐sample MR, mediation MR analysis, and PheWAS to investigate the association between plasma proteins and childhood asthma. Initially, we assessed the potential causal role of 4489 plasma proteins in mediating childhood asthma. Subsequently, four risk factors associated with childhood asthma were identified and their causal relationship with childhood asthma was assessed using MR analysis. Mediation analyses were subsequently conducted to evaluate the indirect effect of plasma proteins on childhood asthma through the identified risk factors. Lastly, comprehensive phenotypic association studies of plasma proteins were conducted using the UK Biobank dataset. MR, Mendelian randomization; PheWAS, phenome‐wide association studies.

In this study, we conducted a two‐sample MR analysis to investigate the causal effects of plasma proteins on childhood asthma. We utilized genetic IVs derived from 4489 circulating plasma proteins and genetic associations of childhood asthma. We evaluated the causal relationships between plasma proteins and risk factors for childhood asthma. Subsequently, we conducted mediation analysis to assess the indirect effects of plasma proteins on childhood asthma via these risk factors. Finally, we conducted a phenome‐wide association study (PheWAS) analysis using UK Biobank data to assess the safety of targeting these proteins for childhood asthma treatment (Figure 1). The MR design relies on three fundamental assumptions. Firstly, the genetic instrument used should demonstrate a significant correlation with the exposure. Secondly, the genetic instruments should only affect the outcome through the exposure. Thirdly, the genetic instruments exhibited no association with the confounding factors of the exposure‐outcome relationship.

### 2.2 Genetic associations with plasma proteins

We obtained GWAS summary statistics for proteins from the MR‐Base NHGRI‐EBI GWAS Catalog (https://gwas.mrcieu.ac.uk/datasets/?gwas_id__icontains=prot‐a, https://gwas.mrcieu.ac.uk/datasets/?gwas_id__icontains=prot‐b, https://gwas.mrcieu.ac.uk/datasets/?gwas_id__icontains=prot‐c) and limited our analysis to datasets available for direct download from the TwoSampleMR R package (https://github.com/MRCIEU/TwoSampleMR), a dedicated tool for MR analysis (Table S1).^12^, ^20^, ^21^ To satisfy the first assumption, we required that the datasets used in our study included genetic variants validated at the genome‐wide level of statistical significance. Finally, we selected 4419 proteins (of 4489) for MR analyses by including only those with at least one single nucleotide polymorphism (SNP) showing an association *p*‐value meeting the genome‐wide significant threshold of *p* < 5.0 × 10^−8^.

### 2.3 Genetic associations with childhood asthma and its risk factors

We conducted an initial search using the keywords “childhood asthma” in the MR‐Base GWAS directory (https://gwas.mrcieu.ac.uk/) to obtain relevant GWAS data for our study. Eventually, we identified a dataset (ukb‐d‐ASTHMA_CHILD) that was specifically associated with childhood asthma phenotypes. To address potential confounding factors due to population stratification, we restricted our analysis to individuals of European ancestry and utilized estimates of the associations between protein IVs and childhood asthma.

### 2.4 Genetic associations of risk factors associated with childhood asthma

The selection of secondary outcomes, namely childhood asthma risk factors, was based on a comprehensive review of the literature. We conducted an extensive search on the Pubmed database using the keywords “childhood asthma” and “risk factors” to identify relevant publications. Subsequently, an exhaustive search was conducted to analyze publicly available GWAS summary statistics pertaining to these risk factors, leading to the identification of four pertinent factors,^22^, ^23^, ^24^, ^25^, ^26^, ^27^ namely mood swings, body mass index (BMI), hay fever or allergic rhinitis, and eczema or dermatitis. The pQTLs used as IVs for the secondary outcomes were the same as those used for the primary outcomes. Whenever possible, we retrieved single‐nucleotide polymorphism (SNP)‐outcome effects for all the aforementioned risk factors from published GWAS.

### 2.5 Mendelian randomization analyses

The analyses were performed using the TwoSampleMR R package version 0.5.6 (https://github.com/MRCIEU/TwoSampleMR).^28^ To ensure the robustness of the exposure association (Assumption 1), only SNPs with genome‐wide significant variants (*p* < 5 × 10^−8^) were included in the MR analysis. In this study, we conducted a clumping process (*R* ^2^ < 0.001 and clumping distance = 10,000 kb) to assess linkage disequilibrium (LD) among the included SNPs, aiming to minimize potential bias introduced by strong LD. Another critical step in MR is to ensure that SNP effects on exposure correspond to the effects on the outcome allele. Palindromic SNPs, such as those with A/T or G/C alleles, were excluded to avoid distortion of strand orientation or allele coding. During the harmonization process, alleles were aligned with the human genome reference sequence (build 37), and any ambiguous or duplicated SNPs were removed. The Inverse‐variance weighted (IVW) method, employing a meta‐analysis technique to combine Wald estimates for each SNP as valid natural experiments, was employed. The IVW method served as the primary analysis in this study to estimate the causal relationships between exposure and outcome. Statistical significance was determined using a threshold of *p* < 0.05.

### 2.6 Mediation analysis

A mediation analysis was performed to assess the effects of proteins that exhibit causal associations with both childhood asthma and risk factors on childhood asthma outcomes, considering the mediating role of the risk factors. The exposure to the outcome manifests as a total effect, comprising both direct effects and indirect effects mediated by one or more factors. The primary MR analysis, commonly referred to as a standard univariable MR analysis, captured the overall effect in this study. The direct and indirect effects were identified by leveraging two‐step MR results and estimating the beta coefficient of the indirect effect using the Product method. The Product of Coefficients method involves estimating two regression models. Firstly, the mediator is regressed on the exposure. Secondly, the outcome is regressed on the mediator, adjusting for the exposure. Then, these two estimates are multiplied to estimate the indirect effect. Furthermore, the Delta method was employed to estimate the standard error (SE) and confidence intervals (CIs) for the indirect effect.^29^

### 2.7 Phenome‐wide association studies

To expand our investigation on the potential side effects associated with the 10 proteins linked to childhood asthma, we conducted Phenome‐wide association studies (PheWAS) encompassing a range of diseases. The effects of SNPs and their impact on outcomes were analyzed using summary statistics, incorporating a sample size of up to 408,961 individuals from the UK Biobank cohort.^30^ GWAS analyses were performed by the researchers employing the SAIGE v.0.29 software, which utilizes a generalized mixed model approach to address potential confounding arising from the imbalanced distribution of cases and controls. Phenotypic outcomes of diseases or conditions were defined using the “PheCodes” system, which organizes codes from the International Classification of Diseases and Related Health Problems (ICD‐9/‐10) corresponding to specific phenotypic manifestations.^31^

## 3 RESULTS

### 3.1 Identification of plasma proteins associated with childhood asthma

**FIGURE 2:**
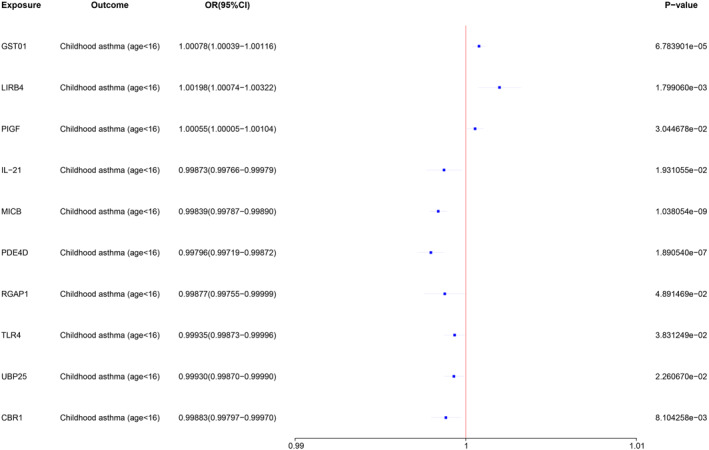
Association between plasma proteins and childhood asthma. MR analysis of the causal effects of 10 plasma proteins on childhood asthma outcome. CI, confidence interval; MR, Mendelian randomization; OR, odds ratio.

We investigated the causal relationship between 4419 (of 4489) plasma proteins and childhood asthma, and identified 10 proteins associated with the risk of childhood asthma by MR analysis (Figure 2 and Table S2). Among these proteins, three increased the risk of developing childhood asthma, whereas the remaining seven proteins decreased it. Glutathione S‐transferase omega‐1 (GSTO1), leukocyte immunoglobulin‐like receptor subfamily B member 4 (LIRB4), and placental growth factor (PIGF) were associated with a greater risk of childhood asthma. On the other hand, interleukin‐21 (IL‐21), Major Histocompatibility Complex class I polypeptide‐related sequence B (MICB), cAMP‐specific 3′,5′‐cyclic phosphodiesterase 4D (PDE4D), Rac GTPase‐activating protein 1 (RGAP1), Toll‐like receptor 4: Lymphocyte antigen 96 complex (TLR4), Ubiquitin carboxyl‐terminal hydrolase 25 (UBP25), and Carbonyl reductase [NADPH] 1 (CBR1) were associated with lower risk of childhood asthma. Specifically, higher genetically predicted levels of LIRB4 (odds ratios [OR] = 1.0020; 95% CI, 1.0007–1.0032; *p* = 1.80 × 10^−3^) were observed to increase the risk of asthma in children. Relatively high genetic prediction levels of MICB (OR = 0.9984; 95% CI, 0.9979–0.9989; *p* = 1.04 × 10^−9^) and PDE4D (OR = 0.9980; 95% CI, 0.9972–0.9987; *p* = 1.89 × 10^−7^) were observed to reduce the risk of asthma in children. The MR sensitivity analyses were consistent with the preliminary analyses, showing no heterogeneity or horizontal pleiotropy.

### 3.2 Identification of likely causal childhood asthma risk factors

We conducted an investigation into the potential causal mechanisms that link plasma proteins to childhood asthma by employing a two‐step mediation MR analysis that accounted for conventional risk factors. In the first step, we examined the causal relationship between plasma proteins and childhood asthma. Subsequently, we analyzed the causal relationship between traditional risk factors for childhood asthma and the onset of the disease. Lastly, we performed mediation analysis to elucidate the impact of plasma proteins on the risk of childhood asthma through traditional risk factors.

**FIGURE 3:**
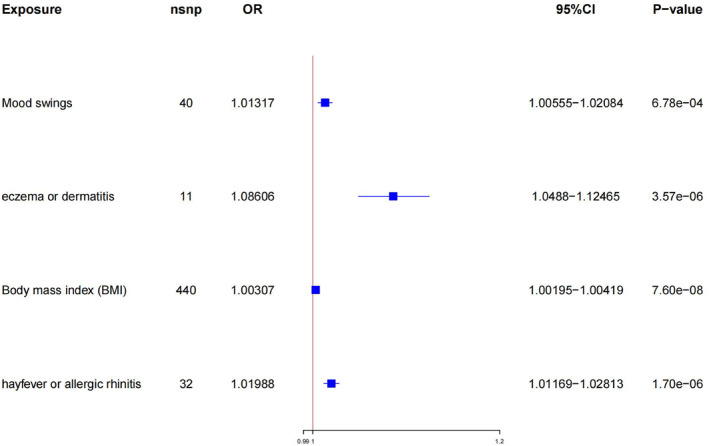
Association between identified risk factors and childhood asthma. MR analysis of the causal effects of risk factors on childhood asthma outcome. CI, confidence interval; MR, Mendelian randomization; OR, odds ratio.

For each of the four childhood asthma risk factors (BMI, mood swings, hay fever or allergic rhinitis, and eczema or dermatitis) we studied, we obtained IVs from publicly available GWAS summary statistics that were based on European populations. Through MR analysis, BMI (OR = 1.0030; 95% CI, 1.0020–1.0042; *p* = 7.6 × 10^−8^), mood swings (OR = 1.0132; 95% CI, 1.0056–1.0208; *p* = 6.8 × 10^−4^), hay fever or allergic rhinitis (OR = 1.0199; 95% CI, 1.0117–1.0281; *p* = 1.70 × 10^−6^) and eczema or dermatitis (OR = 1.0861; 95% CI, 1.0488–1.1246; *p* = 3.57 × 10^−6^) were identified as factors that heightened the risk of childhood asthma (Figure 3 and Table S3).

### 3.3 Identification of childhood asthma risk factors associated proteins

We performed MR analysis on a total of 4419 (of 4489) plasma proteins to assess their associations with four highlighted risk factors for childhood asthma. The findings revealed that 73 proteins exhibited associations with at least one of the childhood asthma risk factors. Specifically, 40 proteins were associated with BMI, 13 proteins with mood swings, and 25 proteins with allergic diseases (Table S4). No heterogeneity or horizontal pleiotropy was detected, and sensitivity analyses produced consistent estimates of causal effects. Notably, we found that a genetically predicted higher PDE4D level was associated with a lower BMI (OR = 0.9669; 95% CI, 0.9546–0.9793; *p* = 2.23 × 10^−7^). Higher levels of genetically determined RGAP1 were associated with a reduced risk of eczema or dermatitis (OR = 0.9963; 95% CI, 0.9942–0.9984; *p* = 5.04 × 10^−4^). The effect directions of the associations between proteins and risk factors were consistent with those between proteins and childhood asthma, suggesting that these risk factors may serve as mediators in the protein‐childhood asthma associations.

### 3.4 Mediation effect of plasma proteins on childhood asthma outcomes via risk factors

**FIGURE 4:**
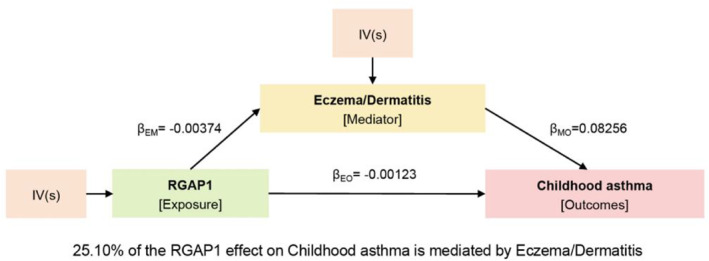
Indirect effects of plasma proteins on childhood asthma through risk factors. Mediation analysis to assess the indirect effects of plasma proteins on childhood asthma through risk factors. *β*_EM_, effects of exposure on mediator; *β*_EO_, effects of exposure on outcome; *β*_MO_, effects of mediator on outcome.

In order to investigate the indirect influence of plasma proteins on childhood asthma outcome through risk factors, we performed a mediation analysis using effect estimates obtained from two‐step MR and the total effect from primary MR. Our analysis focused on four proteins, IL‐21, MICB, PDE4D, and RGAP1, which exhibited effects in both MR analyses involving risk factors and childhood asthma outcomes (Figures 4 and S1–S3). The product method was employed to determine the indirect effect, while the delta method was used to calculate the standard errors (SE) and CIs as described in the Methods section. Our findings indicate that RGAP1 mediates a significant proportion (25.10%) of the risk of childhood asthma through eczema or dermatitis (Figure 4). The indirect effects of MICB, PDE4D, and IL‐21 on childhood asthma via BMI were relatively modest, at 3.31%, 5.03%, and 3.43%, respectively (Figures S1–S3). This suggests that MICB, PDE4D, and IL‐21 plasma proteins do not influence the occurrence of childhood asthma through BMI.

### 3.5 PheWAS analysis of childhood asthma‐associated proteins

**FIGURE 5:**
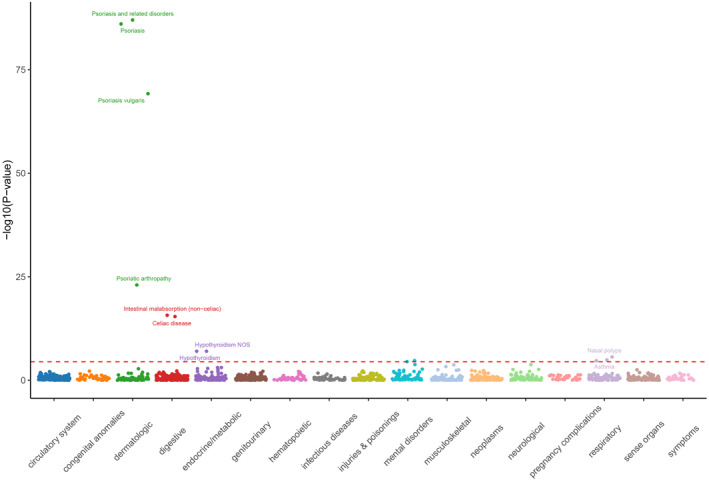
Associations between childhood asthma‐associated proteins and other disease outcomes revealed by PheWAS analysis.

To evaluate the potential effects of the 10 proteins associated with childhood asthma on other medical conditions, we conducted a PheWAS analysis involving 1403 diseases and traits within the UK Biobank dataset. Notably, elevated genetic levels of plasma protein UBP25 not only correlate with a reduced risk of childhood asthma but also with an increased risk of skin diseases such as psoriasis and related disorders, as well as a decreased risk of digestive disorders like intestinal malabsorption and celiac disease, along with endocrine metabolic diseases like hypothyroidism (Figure 5). This suggests that in the future, if plasma protein UBP25 is to be developed as a therapeutic target for childhood asthma treatment, its potential side effects and safety profile must be taken into account. Furthermore, genetically higher plasma levels of IL‐21 and MICB are associated with an elevated risk of digestive disorders such as intestinal malabsorption and endocrine metabolic disorders including Type 1 diabetes, thyrotoxicosis with or without goiter, and Graves' disease. In terms of RGAP1, it demonstrates beneficial effects on the risk of digestive system diseases like intestinal malabsorption and neurological disorders such as multiple sclerosis, while exhibiting deleterious effects on musculoskeletal diseases like rheumatoid arthritis and other inflammatory polyarthropathies as well as polymyalgia rheumatica.

## 4 DISCUSSION

Based on the genetic data of 4419 (of 4489) plasma proteins from UK Biobank, our study presents compelling evidence linking 10 plasma proteins (GSTO1, LIRB4, PIGF, IL‐21, MICB, PDE4D, RGAP1, TLR4, UBP25 and CBR1) to childhood asthma. Our findings demonstrated a causal association of mood swings, hay fever or allergic rhinitis, and eczema or dermatitis with childhood asthma risk. These risk factors played a significant role in the development of childhood asthma, aligning with established epidemiological data. Furthermore, we identified 73 proteins that were causally linked to these risk factors. Additionally, the PheWAS‐MR analysis unveiled potential beneficial indications for therapeutically targeting the 10 childhood asthma‐associated proteins, while also highlighting a few safety concerns.

Glutathione S‐transferase omega‐1 (GSTO1), an omega‐class GST (Glutathione S‐transferase), is crucial in regulating the S‐sulfation status of intracellular components involved in cancer cell viability and inflammatory processes.^32^ An observational study identified a notable upregulation of GSTO1 expression in neutrophils obtained from individuals diagnosed with chronic obstructive pulmonary disease (COPD).^33^ Another independent study further established a correlation between GSTO1 and the severity of inflammation and respiratory dysfunction in patients with cystic fibrosis.^34^ However, despite extensive research, a limited number of studies have successfully established a definitive association between GSTO1 and asthma. This study uncovers a significant and causal association between GSTO1 and childhood asthma, where increased levels of genetically determined GSTO1 are linked to an elevated risk of developing this condition.^35^, ^36^, ^37^ Other studies have demonstrated that inhibiting GSTO1‐1 using shRNA in LPS‐exposed macrophages reduces the activation of interleukin (IL)‐1β, IL‐6, and NLRP3 inflammasomes.^38^ Additionally, using GSTO1‐1‐deficient mice in inflammatory disease models supported these in vitro findings, showing decreased expression levels of IL‐1β, IL‐6, and TNF‐α in LPS‐induced inflammation compared to wild‐type (WT) controls. These findings suggest a potential role of GSTO1 in promoting asthma development through the induction of inflammatory responses and damage to airway epithelial cells. Conversely, inhibiting GSTO1 seems to offer benefits in reducing the severity of the disease.

Phosphodiesterase 4D (PDE4D) is a protein encoded by the PDE4D gene and has been associated with multiple diseases, including asthma, COPD, stroke and bone mineral density.^39^, ^40^, ^41^ Genome‐wide association analysis demonstrated that PDE4D SNPs, specifically rs1588265 and rs1544791, exhibited protective effects against childhood asthma.^42^ The summary OR for these SNPs were 0.85 (95% CI: 0.77, 0.93) and 0.85 (95% CI: 0.78, 0.94), respectively, which align with our own findings. However, studies have demonstrated that cyclic adenosine monophosphate (cAMP) can inhibit various inflammatory responses, whereas PDE4D, which can specifically hydrolyze cAMP, may exacerbate the inflammatory response.^43^ This divergence in protein function could potentially be attributed to age‐related factors. Consequently, a larger sample size is necessary to obtain additional support for investigating the role of PDE4D in asthma, specifically in the pediatric population.

Phosphatidylinositol glycan anchor biosynthesis class F (PIGF) is a protein involved in the biosynthesis of glycosylphosphatidylinositol (GPI) anchors.^44^ The GPI‐anchor is a glycolipid containing three mannose molecules in its core backbone. It is found in many blood cells, where it functions to anchor proteins to the cell surface. Studies have reported higher levels of PIGF protein in children with atopic dermatitis (AD).^45^ There are clear epidemiological parallels between asthma and AD. Importantly, AD frequently manifests as the initial symptom of an atopic diathesis, which affects genetically predisposed individuals and also encompasses asthma and allergic rhinitis. Up to 80% of children with AD eventually develop allergic rhinitis or asthma later in childhood.^46^ This is consistent with the study's findings, which observed that higher genetically predicted levels of PIGF (OR = 1.0005; *p* = 0.0304) increased the risk of asthma in children. This suggests that the PIGF protein may serve as a potential therapeutic target for treating childhood asthma. Future research necessitates larger sample sizes and more comprehensive cellular experiments and animal models to validate the therapeutic efficacy of PIGF protein in the treatment of childhood asthma.

A range of sensitivity analyses was performed to uphold the validity of the IVs used in the MR analysis and to ensure compliance with the underlying assumptions. To select the instruments for each plasma protein level, LD clumping was employed with a threshold of *R* ^2^ < 0.001 for plasma proteins displaying a significance level of *p* < 5 × 10^−8^. Subsequently, the IVs used in the MR analysis underwent comprehensive evaluation for heterogeneity, horizontal pleiotropy, and robustness. Notably, no significant heterogeneity and horizontal pleiotropy was observed (*p* > 0.05), and the robustness of the IVs was corroborated through a leave‐one‐out sensitivity test.

The present study exhibits notable strengths. This study employs a GWAS with a substantial sample size, enabling a thorough analysis of plasma proteins in relation to childhood asthma occurrences. In addition, a comprehensive range of pleiotropy assessment and sensitivity analyses were conducted, which relaxed IV assumptions and bolstered the robustness of our MR findings.

We conducted a comprehensive investigation of the DrugBank database to assess the druggability of candidate protein targets.^47^ Interestingly, we discovered that several proteins identified in the MR analysis are druggable, including PDE4D, TLR4, and CBR1. Notably, there are drugs targeting PDE4D, such as Cilomilast, developed for the treatment of respiratory disorders, such as asthma and COPD. Cilomilast is orally active and functions as a selective phosphodiesterase‐4 inhibitor. Therefore, PDE4D holds great potential as a drug target for childhood asthma treatment. However, although drugs targeting TLR4 and CBR1 proteins have been developed, such as TLR4 (Papain and Cyclobenzaprine) and CBR1 (Heptaethylene glycol and Haloperidol), no drugs have been found for treating childhood asthma. Future research may be required.

Moreover, the indirect effects of MICB, PDE4D, and IL‐21 on childhood asthma via BMI are relatively modest, at 3.31%, 5.03%, and 3.43%, respectively. This suggests that MICB, PDE4D, and IL‐21 proteins do not influence the occurrence of childhood asthma through BMI. Luigi Tortola et al. found that IL‐21 production by TH2 and follicular helper T/ex‐follicular helper T cells promotes asthma by inhibiting Treg cells.^48^ Hence, the specific mechanisms by which MICB, PDE4D, and IL‐21 proteins affect the onset of childhood asthma will require further experimental evidence for elucidation.

However, there are a few limitations to this study. First, participant overlap between exposure and outcome measurements could compromise the data quality. Second, the lack of Bonferroni correction for *p*‐values in the MR analysis suggests potential false positive results, underscoring the need for further experimental validation of the findings. Lastly, the study solely relied on GWAS data pertaining to plasma protein levels, lacking information on tissue‐specific proteins, particularly those from lung tissue. Further investigation of lung tissue‐associated candidate proteins may enhance the reliability and robustness of experimental outcomes.

Our results highlight potential targets for future therapies aimed at improving childhood asthma outcomes, demonstrating the relevance of proteomics in identifying drug targets. Further research is needed to evaluate the viability of the 10 identified proteins as potential targets for childhood asthma treatment. As more comprehensive proteomics platforms become available and non‐European ancestry populations are included in studies, additional drug targets may be discovered.

## 5 CONCLUSION

In this study, we conducted a causal analysis to investigate the relationship between plasma proteins and childhood asthma, and its risk factors. Additionally, we performed correlation analyses using data from the UK Biobank to assess the associations between 10 plasma proteins and various other diseases.

## AUTHOR CONTRIBUTIONS

The authors' responsibilities were as follows—Shang‐Qin Chen, Xiu‐Feng Huang, Zhen‐Lang Lin: study conception and design; all authors: data acquisition and analysis; Yi‐Qing Wu, Yi‐Xin Cai, Xiao‐Li Chen, Xiu‐Feng Huang: drafting the manuscript and figures; all authors: reviewing the manuscript; and all authors: read and approved the final manuscript.

## CONFLICT OF INTEREST STATEMENT

The authors report no conflicts of interest.

## Supporting information

Supporting Information S1

Supporting Information S1

## Data Availability

All data utilized in this study were sourced from publicly released GWAS summary statistics. Childhood asthma (age < 16): https://gwas.mrcieu.ac.uk/datasets/ukb‐d‐ASTHMA_CHILD/. Plasma proteins: https://gwas.mrcieu.ac.uk/datasets/?gwas_id__icontains=prot‐a, https://gwas.mrcieu.ac.uk/datasets/?gwas_id__icontains=prot‐b, https://gwas.mrcieu.ac.uk/datasets/?gwas_id__icontains=prot‐c. Body mass index (BMI): https://gwas.mrcieu.ac.uk/datasets/ukb‐b‐19953/. Doctor diagnosed hay fever or allergic rhinitis: https://gwas.mrcieu.ac.uk/datasets/ukb‐b‐7178/. Non‐cancer illness code self‐reported: eczema/dermatitis: https://gwas.mrcieu.ac.uk/datasets/ukb‐a‐99/. Mood swings: https://gwas.mrcieu.ac.uk/datasets/ukb‐a‐45/. All datasets generated in this study are included in the article/supplementary materials.
