# Supplementary material for: Proteomic analysis reveals potential therapeutic targets for childhood asthma through Mendelian randomization
Source: Clin Transl Allergy. 2024 May 10;14(5):e12357. doi: 10.1002/clt2.12357 (PMC11087394; doi:10.1002/clt2.12357)

**List of Supplementary Data**

**Figure S1-S3.** Indirect effect of plasma proteins on childhood asthma via risk factors.

**Figure S1.** Indirect effect of plasma proteins on childhood asthma via risk factors.

Indirect effect of MICB on childhood asthma through BMI. βEM, effects of exposure on mediator; βMO, effects of mediator on outcome; βEO, effects of exposure on outcome.


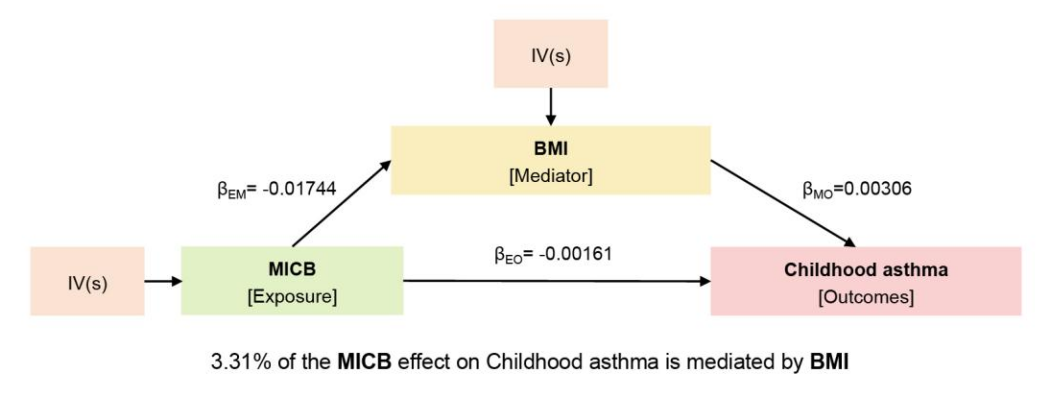


**Figure S2.** Indirect effect of plasma proteins on childhood asthma via risk factors.

Indirect effect of PDE4D on childhood asthma through BMI. βEM, effects of exposure on mediator; βMO, effects of mediator on outcome; βEO, effects of exposure on outcome.


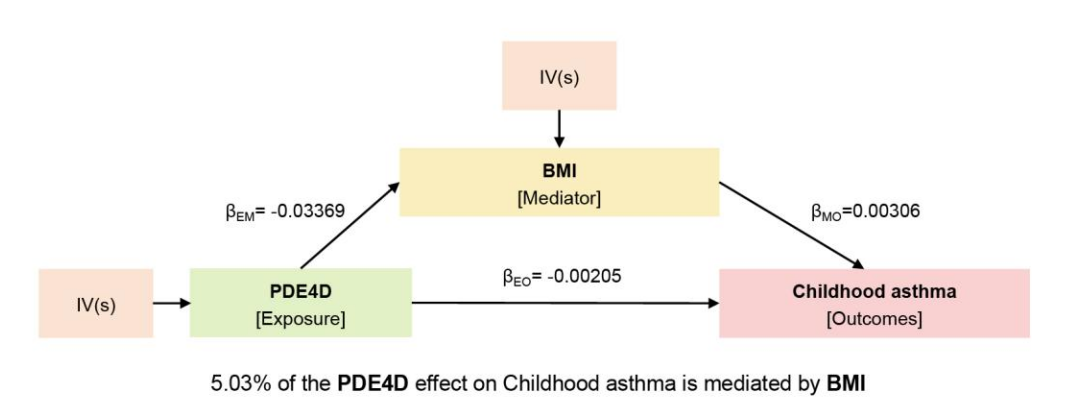


**Figure S3.** Indirect effect of plasma proteins on childhood asthma via risk factors.

Indirect effect of IL-21 on childhood asthma through BMI. βEM, effects of exposure on mediator; βMO, effects of mediator on outcome; βEO, effects of exposure on outcome.


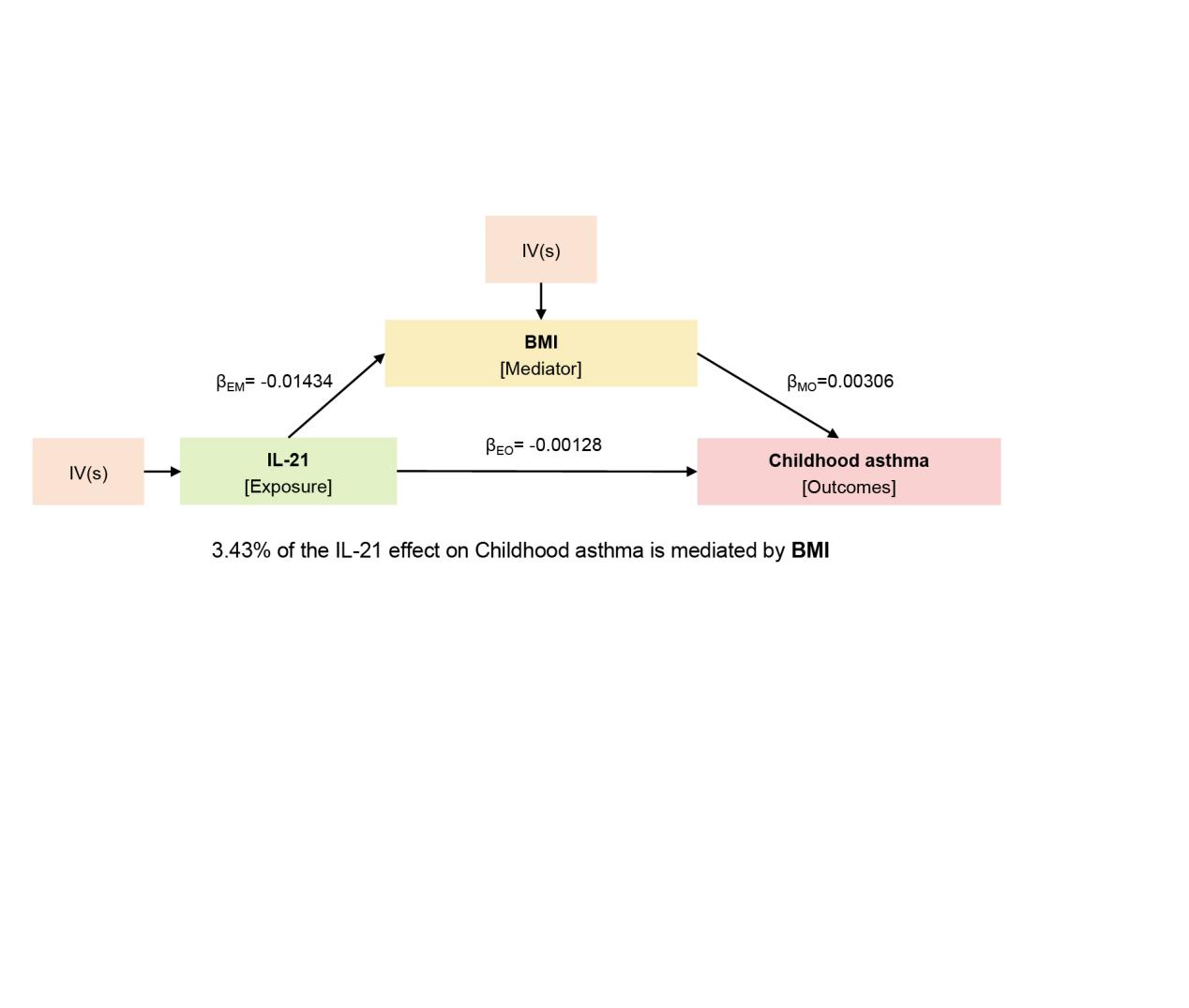

Supplement: Supplementary file 1 — Supporting Information S1 [file CLT2-14-e12357-s002.docx]
